# Inadvertent intrathecal injection of tranexamic acid

**DOI:** 10.4103/1658-354X.76504

**Published:** 2011

**Authors:** Olfa Kaabachi, Mongi Eddhif, Karim Rais, Mohamed Ali Zaabar

**Affiliations:** *Department of Anaesthesiology and Intensive Care, Kassab Orthopaedic Institute of Tunis, Tunisia*

**Keywords:** *Intrathecal*, *seizures*, *tranexamic acid*, *ventricular tachycardia*

## Abstract

Some factors have been identified as contributing to medical errors such as labels, appearance, and location of ampules. In this case report, inadvertent intrathecal injection of 80 mg tranexamic acid was followed by severe pain in the back and the gluteal region, myoclonus on lower extremities and agitation. General anesthesia was induced to complete surgery. At the end of anesthesia, patient developed polymyoclonus and seizures needing supportive care of the hemodynamic, and respiratory systems. He developed ventricular tachycardia treated with Cordarone infusion. The patient’s condition progressively improved to full recovery 2 days after. Confusion between hyperbaric bupivacaine and tranexamic acid was due to similarities in appearance between both ampules.

## INTRODUCTION

In industrialized countries, it has been reported that adverse events from drugs are a leading cause of injury and death. Some factors have been identified as contributing to medical errors such as labels, appearance and location of ampules and syringes, the lack of double-checking, inattention, poor communication, and fatigue on the part of the anesthesiologist.[1,2] Tranexamic acid (TXA) is being widely used during surgery to reduce bleeding and has been associated with previous accidental intratechal injection with fatal issue in some cases.[3–7] In this case report, the injection of TXA instead of hyperbaric bupivacaine for spinal anesthesia was due to confusion between two different ampules with similar appearance, and was responsible of polymyoclonus, seizure, and ventricular arrhythmia.

## CASE REPORT

**Figure 1:**
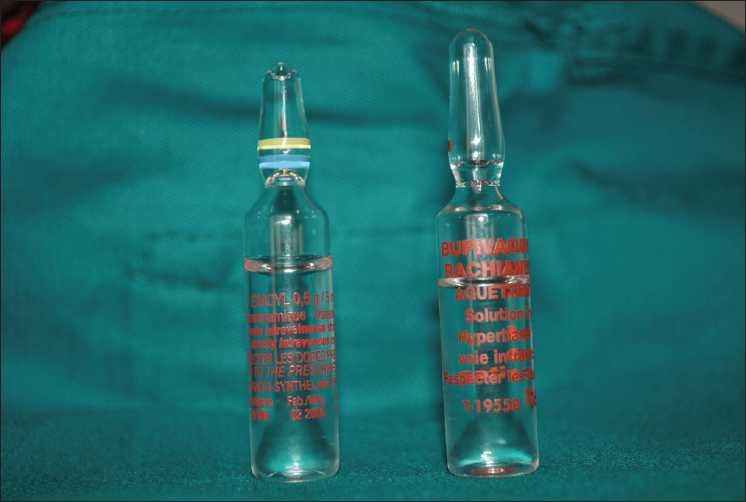
TXA and old bupivacaine ampules

**Figure 2:**
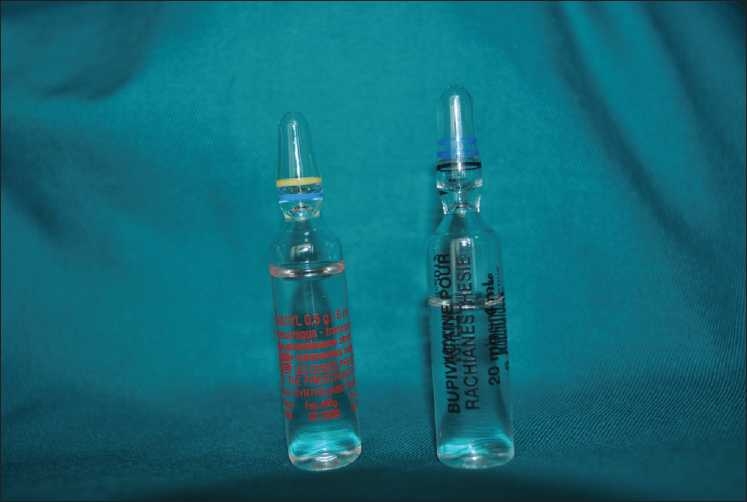
TXA and new bupivacine ampules

A 30-year-old man, ASA I physical status, was scheduled for arthroscopic knee anterior ligament reconstruction. Spinal anesthesia was performed with the patient in the sitting position at the L4--L5 interspace, using a 26-gauge Quincke tip needle. About 60 s after injection of 8 mg (1.6 ml) of 0.5% hyperbaric bupivacaine, the patient complained of severe pain in the back and the gluteal region and developed myoclonic movements in the lower extremities. The patient’s arterial blood pressure increased to 160/100 mmHg and the heart rate to 120 beats/min. IV sedation with midazolam (3 mg) and fentanyl (100 μg) was administered immediately without effect. Consequently, general anesthesia was induced by the infusion of propofol (200 mg) and celocurine (100 mg), and the patient’s trachea was intubated. Anesthesia was maintained using propofol infusion (10 mg/kg/h) and Fentanyl (2 μg/kg/45 min) and surgery was continued. At the end of surgery and 5 mins after the propofol infusion was discontinued, recurrent polymyoclonus and seizures developed. Clonazepam (1 mg) and phenobarbital (800 mg) were administered and continuous sedation using midazolam and fentanyl was started. Accidental intrathecal injection of the wrong drug was suspected and a used ampule of TXA was found in the trash can [Figures 1 and 2]. The patient was transferred to the intensive care unit about 2 h after the injection and mechanical ventilation with volume controlled ventilation mode was continued. Central venous and arterial lines were inserted to the patient. The first postoperative arterial blood gas analysis revealed metabolic acidosis (pH = 7.3, PaO_2_ = 183, PaCO_2_ = 35, HCO_3_^-^ = 17.7). The blood analysis did not reveal any renal, hepatic, or hematological failure. The patient experienced tonico-clonic convulsions in the upper extremities and the face 3 h postoperatively, which were treated by an infusion of sodium thiopental (3–5 mg/kg/h). Gastric administration of Phenobarbital 200 mg daily was initiated. Cranial computed tomography was without abnormalities.

A prophylactic infusion of cordarone at the rate of 10 mg/kg/24 h was administered for 24 h, in order to prevent ventricular arrhythmia. The patient developed several episodes of ventricular tachycardia that regressed spontaneously.

On the second postoperative day, the sedation was arrested. The patient’s level of consciousness increased. He moved his head and upper extremities with painful stimulus, deep tendon reflexes were absent in the lower extremities. On the third postoperative day, he opened his eyes to voice commands, obeyed to simple orders and breathe spontaneously. Tracheal was extubated. Finally, on the fourth postoperative day, all neurologic examinations were normal. The patient was discharged from the intensive care unit and on the sixth day was subsequently discharged from the hospital without neurological sequelae. Six months later, neurological evaluation was still normal.

## DISCUSSION

Little is known about the effect of direct intrathecal administration of TXA. Wong *et al*.[3] reported the first case of inadvertent intrathecal injection of 75 mg TXA in an ASA I physical status, 18-year-old man, scheduled for appendectomy. Four hours after the spinal injection, he experienced persistent motor and sensory block of lower extremities and urinary incontinence. He developed clonic convulsions that progressed to a generalized seizure and hyperthermia of 40.5°C 5.5 h after the injection. His seizure and fever gradually subsided over the next 5 h after treatment with intravenous diazepam and diclofenac and the patient recovered completely, without any sequelae.

De Leede-Van der Maarl *et al*.[4] reported a case of a 68-year-old man who accidentally received an intrathecal injection of 50 mg TXA. Immediately after the administration of the drug, he developed status epilepticus. The outcome was complicated, with hypotonic paresis of all four extremities, which resolved but resulted in residual bilateral peroneal palsy. In the case reported by Yeh *et al*.,[5] generalized convulsions and refractory ventricular fibrillation after intrathecal administration of 500 mg of TXA was associated with fatal outcome. In two others case reports, intrathecal injection of a dose of 150 mg of TXA lead to a poor issue in one case because of a refractory ventricular fibrillation.[6,7] Our patient received an intrathacal injection of 90 mg TXA with a full recovery 4 days after.

The exact mechanism by which TXA induces convulsions or ventricular arrhythmia is unknown. High doses of TXA would cause massive sympathetic discharge, as evidenced by the initial hypertensive response and the subsequent ventricular arrhythmia reported in our case report and also in some patient.[4,5,7] TXA-induced seizures either from direct cerebral ischemia secondary to decreases in regional or global cerebral blood flow,[8] or blockage of inhibitory cortical-aminobutyric acid (GABA)-A receptors.[9] There is evidence for a dose-related neurotoxicity in animal model, with greater severity and duration of seizure with increasing TXA dosage.[10] Use of high-dose TXA in patients undergoing cardiac surgery increased at the incidence of convulsive seizures from 1.3 to 3.8%.[11]

Finally, all theses case reports were due to confusion between hyperbaric bupivacaine and TXA. The ampules of TXA (100 mg/mL) and bupivacaine (5 mg/mL) were similar in appearance [Figure 1]. But, all these publications have leaded the manufacturer to change ampule of hyperbaric bupivacaine [Figure 2], so such serious complication could not happen again.
